# Characteristics of Infection Immunity Regulated by *Toxoplasma gondii* to Maintain Chronic Infection in the Brain

**DOI:** 10.3389/fimmu.2018.00158

**Published:** 2018-02-05

**Authors:** Young Sang Hwang, Ji-Hun Shin, Jung-Pyo Yang, Bong-Kwang Jung, Sang Hyung Lee, Eun-Hee Shin

**Affiliations:** ^1^Department of Parasitology and Tropical Medicine, Seoul National University College of Medicine, and Institute of Endemic Diseases, Seoul National University, Seoul, South Korea; ^2^Institute of Parasitic Diseases, Korea Association of Health Promotion, Seoul, South Korea; ^3^Department of Neurosurgery, Seoul National University College of Medicine, SMG-SNU Boramae Medical Center, Seoul, South Korea; ^4^Seoul National University Bundang Hospital, Seongnam, South Korea

**Keywords:** *Toxoplasma gondii*, brain, chronic infection, microglial polarization, infection immunity

## Abstract

To examine the immune environment of chronic *Toxoplasma gondii* infection in the brain, the characteristics of infection-immunity (premunition) in infection with *T*. *gondii* strain ME49 were investigated for 12 weeks postinfection (PI). The results showed that neuronal cell death, microglia infiltration and activation, inflammatory and anti-inflammatory cytokine expression, Stat1 phosphorylation, and microglia activation and inflammatory gene transcripts related to M1 polarization in the brain were increased during the acute infection (AI) stage (within 6 weeks PI), suggesting that innate and cellular inflammatory response activation and neurodegeneration contributed to excessive inflammatory responses. However, these immune responses decreased during the chronic infection (CI) stage (over 6 weeks PI) with reductions in phosphorylated STAT1 (pSTAT1) and eosinophilic neurons. Notably, increases were observed in transcripts of T-cell exhaustion markers (TIM3, LAG3, KLRG1, etc.), suppressor of cytokines signaling 1 protein (SOCS1), inhibitory checkpoint molecules (PD-1 and PD-L1), and Arg1 from the AI stage (3 weeks PI), implying active immune intervention under the immune environment of M1 polarization of microglia and increases in inflammatory cytokine levels. However, when BV-2 microglia were stimulated with *T. gondii* lysate antigens (strain RH or ME49) *in vitro*, nitrite production increased and urea production decreased. Furthermore, when BV-2 cells were infected by *T. gondii* tachyzoites (strain RH or ME49) *in vitro*, nitric oxide synthase and COX-2 levels decreased, whereas Arg1 levels significantly increased. Moreover, Arg1 expression was higher in ME49 infection than in RH infection, whereas nitrite production was lower in ME49 infection than in RH infection. Accordingly, these results strongly suggest that immune triggering of *T. gondii* antigens induces M1 polarization and activation of microglia as well as increase NO production, whereas *T*. *gondii* infection induces the inhibition of harmful inflammatory responses, even with M1 polarization and activation of microglia and Th1 inflammatory responses, suggesting a host–parasite relationship through immune regulation during CI. This is a characteristic of infection immunity in infection with *T. gondii* in the central nervous system, and SOCS1, a negative regulator of toxoplasmic encephalitis, may play a role in the increase in Arg1 levels to suppress NO production.

## Introduction

*Toxoplasma gondii* is an Apicomplexan pathogen of the central nervous system (CNS) (1). Human *T*. *gondii* infection generally occurs *via* ingestion of oocysts (an environmentally resistant form) released in cat feces or undercooked meat containing tissue cysts (1). Following ingestion, bradyzoites and sporozoites released from cysts and oocysts invade intestinal cells, where they are converted to tachyzoites, which can then be disseminated *via* the blood or lymphatic system to remote organs and can induce an acute infection (AI) or chronic infection (CI) (1). In general, the brain is the most commonly affected site *via* congenital transmission and subsequent CI, which elicits life-long immunity against toxoplasmosis (1). Immune responses to *T. gondii* infection differ during the proliferative (acute) and dormant (chronic and latent) stages and are dependent on differences in phenotype, virulence, and clinical sequelae of the strains of the clonal lineages, such as the highly virulent strain RH (type I) and the avirulent strain ME49 (type II) (1–3). CI of a type II parasite is maintained by the conversion of tachyzoites into bradyzoites, which produce intracellular tissue cysts. The onset and progression of *T. gondii* encystation result from both intrinsic preprogramming within the parasite and the immune response of the host, which eventually help to maintain a CI (1).

The AI stage of the RH strain is characterized by marked elevations in serum Th1 cytokine levels, such as interferon (IFN)-γ, tumor necrosis factor (TNF)-α, interleukin (IL)-12, and IL-18, and is followed by a lethal outcome in mice at 8 days postinfection (PI) (2). In contrast, non-lethal infection (ME49 strain) is characterized by modest elevations in Th1 cytokines that lead to control of *T. gondii* infection and minimal damage to the host (2). More specifically, in the CNS, IFN-γ plays a critical role in the prevention of toxoplasmic encephalitis (TE) during the late stage of infection in mice *via* inhibition of tachyzoite proliferation. However, simultaneous IFN-γ activation of microglia may cause tissue injury *via* the production of toxic metabolites, such as nitric oxide (NO) (4, 5). However, neurodegeneration does not commonly occur during CI of *T*. *gondii*, despite potential NO toxicity (4), possibly to control parasitic proliferation and avoid tissue damage in the infected brain *via* regulation of the appropriate induction between the cytokines IFN-γ, IL-10, and transforming growth factor beta (TGF-β), and the toxic mediator NO (4, 6–8).

Microglia, which are a type of glial cell and account for 10–15% of all cells within the brain parenchyma, are macrophages that reside in the brain and spinal cord, and have plasticity due to the CNS immune environment and consequently play a key role the regulation of CNS inflammatory reactions, tissue injury, and tissue homeostasis (9). During *T. gondii* infection, microglia are major effector cells that prevent NO-mediated pathogen proliferation (5). During the immune response of the host, *T. gondii* infection progresses from an acute stage, where tachyzoites replicates for 2 months PI, to the chronic stage, which is characterized by the formation of dormant cysts after 2 months PI (4, 10, 11). Simultaneously, life-threatening toxoplasmosis characterized by encephalitis is gradually decreased in the latent stage of *T. gondii* infection (7). However, the underlying switching mechanisms from the prevention of pathogen proliferation to the inhibition of cellular toxic immune response in the CNS remain unclear, although previous *in vitro* studies have reported a fractional infection period (1–8, 12–15). For example, the cytokines IL-2, IL-12, IFN-γ, and TNF-α, which inhibit the growth of *T. gondii*, and IL-4, IL-10, and TGF-β, which are involved in the downregulation of the intracerebral immune response, favor the growth of *T*. *gondii* and have been implicated in TE (7). Besides, several reports insisted that the decrease in the expression levels of the effector molecules IFN-γ and NO is important for latent infection of *T. gondii* (4, 5, 8, 15, 16). Hence, active intervention may be a good strategy for prolonged parasitic survival and establishment of a host–parasite relationship without expulsion of the parasites by the host cells.

Our previous study found that a decrease in NO in Tg2576 mice caused Alzheimer’s disease even with inflammation of the brain due to TE (8). In particular, because brain tissue is susceptible to the noxious effects of NO, such as wide-spread neurodegeneration, NO production in *T. gondii* infection has been widely studied as an important regulator and indicator of both protective effects and tissue damage (4, 5, 8, 15, 16). In the CNS, microglial cells lead to broad range of immunoregulatory functions with regulation of NO production to prevent further *T*. *gondii* proliferation (1, 5, 8, 15). However, despite numerous investigations, the time-specific kinetics of microglia activation in chronically infected normal mice have not been defined. Hence, further studies are needed because *T. gondii* infection is very chronic in immunocompetent hosts and can recur with time. Unfortunately, no previous study has investigated the immunomodulation in the brain during long-term CI of *T. gondii* and the key events in the regulation of CI, immunity, and parasite proliferation must be addressed to reduce the harmful effects of CI to brain tissues. As previously revealed, IFN-γ, TNF-α, and IL-12 were found to inhibit parasitic growth both *in vivo* and *in vitro*, and IL-10 and TGF-β are important to reduce the excessive inflammatory response of the brain to CI of *T*. *gondii* (2–7, 12, 15). The interactions of these cytokines inhibit NO production and promote the conversion of M1-type microglial cells to the M2-type *via* alternative activation accompanying arginase 1 (Arg1) activity and expression of the mannose receptor type C (CD206) (3, 5, 7, 12, 15, 16). The effector molecules of *T. gondii* that induce the polarization of macrophages include *Toxoplasma* rhoptry kinase ROP16 in the alternative activation pathway and *Toxoplasma* dense granule protein GRA15 in the classical activation pathway, which activate STAT6 and NF-κB, respectively (3). However, immune modulation may not be limited by the molecular actions of *T. gondii*, but rather may be the result of interactions between the host and parasite to continually change the overall immune response during the latent infection stage due to changes in the immune characteristics and the course of infection. For example, several studies have investigated immune characteristics *via* genetic manipulation, such as the dense granule protein GRA15-KO-T of transgenic *T. gondii* (*Toxoplasma*-Cre) in IL-10^−/−^ mice, but neglected to specify the time-varying infectious immunity during CI in regard to the host–parasite interactions (14, 17–19).

The characteristics of infectious immunity in the *T. gondii*-infected brain have been mainly investigated in TE with a focus on the AI stage (20). The most important effector molecules of TE and protection against *T. gondii* include the IL-12/IFN-γ axis, as well as IFN-γ through signal transducer and activator of transcription 1 (STAT1) and inducible nitric oxide synthase (iNOS) (5, 12, 17). Nevertheless, prolonged *T. gondii* infection results in decreased neurodegeneration and inflammatory immune responses, thus we previously identified components of basic and integrative infection immunity (8). In this respect, we aimed to investigate the processes underlying changes in infection immunity from the AI stage to the CI stage, and identified key responses in the immune regulation between a harmful inflammatory response and restoring neurodegeneration in the brain. To this end, we investigated the changes underlying neurodegeneration during *T. gondii* infection, as well as the activation and polarization of microglial cells as effector cells against *T. gondii* infection. Also, inflammatory and anti-inflammatory responses, changes in transcript expression patterns during the infection period, induction of NO and Arg1, an arginine hydrolytic enzyme, and finally *in vitro* infection of *T. gondii* tachyzoites (RH− or ME49 strain) as well as *in vitro* stimulation of *T. gondii* lysate antigen (TLA) (RH− or ME-TLA) were investigated to reveal the differences in immune-triggering events between *T. gondii* infection in the host–parasite relationship and immune characteristics of TLA as pathogen-associated molecular pattern molecules (PAMPs). The results of the present study are very important and interesting in regard to characterizing the infection immunity processes regulated by the host–parasite relationship through proper control of the protective inflammatory immune response, while reducing the harmful effects of neurodegeneration.

## Materials and Methods

### Experimental Animals

Male, 7-week-old, C57BL/6 mice were purchased from the ORIENT BIO Animal Center (Seongnam, South Korea) and housed at room temperature (20–23°C) on a 12-h light/dark cycle with *ad libitum* access to sterilized (irradiated and autoclaved) food and water. All animal experiments were conducted in accordance with the ethical standards of the Institutional Animal Care and Use Committee of Seoul National University (SNU-110315-5).

### Ethics Statement

This study protocol was approved by the Ethics Committee of Seoul National University and conducted in strict accordance with the Guidelines for Animal Experiments (SNUIBC-R110302-1). All surgeries were performed under anesthesia and all efforts were made to ensure minimal animal suffering.

### *T. gondii* Infection

C57BL/6 mice (Orient Bio) at the age of 7 weeks were intraperitoneally injected with *T. gondii* strain ME49 (infection dose of 10 cysts) isolated from *T*. *gondii*-infected brains harvested at 3 months PI and housed in the animal facility of Seoul National University College of Medicine. Most experiments were conducted at 3 months PI. Six mice were randomly sacrificed at 0, 3, 6, 9, and 12 weeks PI, and brain tissues were collected for histological examinations, cytokine analysis, and microarray analysis.

### Neuronal Degeneration in the Hippocampal Dentate Gyrus (DG) Detected by Hematoxylin and Eosin (H&E) Staining

Brain tissues were embedded in paraffin and coronal-sectioned at thickness of 10 µm through the hippocampus, mounted, and stained with H&E. Then, the tissue specimens were dehydrated with a graded alcohol series, cleared in xylene, fixed with Canadian balsam (Caedax; Merck, Darmstadt, Germany), and mounted on cover slips. Neuronal degeneration in the hippocampal DG was determined by the detection of eosinophilic neurons under a light microscope (Olympus PM-20; Olympus Corporation, Tokyo, Japan). The number of degenerative cells were counted in photomicrographs obtained with a digital camera (Leica DFC 280, Leica Microsystems, Wetzlar, Germany) attached to a microscope (BX-51; Olympus Corporation) using Image J software ver. 1.46.^1^

### Detection of *T. gondii* Cyst in the Brain by Reverse Transcription Polymerase Chain Reaction (RT-PCR)

Total RNA was isolated from *T. gondii-*infected mouse brain tissue using the RNeasy Mini kit (Qiagen, Hilden, Germany) according to the manufacturer’s protocol. All samples were subjected to RT-PCR using the RT premix reverse transcription kit (Elpis Biotech Inc., Daejeon, South Korea) and MG Taq DNA polymerase (Macrogen, Seoul, South Korea) with the following primer pairs: GAPDH (Primer Bank ID 6679937a1): forward 5′-AGG TCG GTG TGA ACG GAT TTG-3′ and reverse 5′-TGT AGA CCA TGT AGT TGA GGT CA-3′ (123 bp); *T*. *gondii* B1 gene: forward 5′-TCC CCT CTG CTG GCG AAA AGT-3′ and reverse 5′-AGC GTT CGT GGT CAA CTA TCG ATT G-3′ (98 bp). RT-PCR was performed for 35 cycles with an annealing temperature of 54–55°C and the products were analyzed by 1% agarose gel electrophoresis.

### Real-time PCR

Total RNA was isolated from Total RNA was isolated from T. gondii-infected mouse brain tissue using the RNeasy Mini kit (Qiagen, Hilden, Germany) according to the manufacturer’s protocol. All samples were reverse transcribed using RT premix (Elpis Biotech Inc.). Real-time PCR was performed using the CFX96 (Bio-Rad) and SYBR green (Enzynomics™, Daejeon, Korea) was used to detect amplification products. The reaction conditions used were; initial denaturation at 95°C for 15 min, 40 amplification cycles [denaturation at 95°C for 10 s, annealing at 60°C for 20 s, and extension at 72°C for 30 s], followed by melting curve analysis. Data analysis was performed using CFX96 software (Bio-Rad). The following primer sequences were used for real-time PCR: GAPDH: forward 5′-GGT GAA GGT CGG TGT GAA CG-3′ and reverse 5′-CTC GCT CCT GGA AGA TGG TG-3′ (578 bp); and Socs1: forward 5′-CTG CGG CTT CTA TTG GGG AC-3′ and reverse 5′-AAA AGG CAG TCG AAG GTC TCG-3′ (217 bp); and Nos2 (iNOS): forward 5′-CAG CAC AGA ATG TTC CAG AAT CC-3′ and reverse 5′-TGT CAT GCA AAA TCT CTC CAC TGC-3′ (105 bp); and Arg1: forward 5′-CTT TAA CCT TGG CTT GCT TCG GAA-3′ and reverse 5′-CTT AGT TCT GTC TGC TTT GCT GTG-3′ (140 bp).

### Immunostaining of *T. gondii*-Infected Mouse Brain Tissues

Mice were sacrificed by CO_2_ asphyxiation at the pre-determined times and brain tissues were collected, then fixed in formalin and embedded in paraffin using the Leica TP1020 Tissue Processor (Leica Microsystems GmbH, Wetzlar, Germany). For specific immunostaining, samples were immunostained using the ChromoMap Kit and the Discovery XT automated staining instrument (Ventana Medical Systems, Inc., Tucson, AZ, USA). Briefly, 4 μm-thick tissue sections were fixed on Probe-On-Plus Slides (Thermo Fisher Scientific, Swedesboro, NJ, USA), deparaffinized in xylene, rehydrated in a graded series of alcohol (100, 95, 80, and 70), and finally rinsed with distilled water. After antigen retrieval, the endogenous peroxidase activity of the samples was blocked by treatment of H_2_O_2_ with blocking buffer [1% fetal bovine serum in phosphate-buffered saline (PBS)] for 30 min. Then, the samples were incubated for 60 min at room temperature with either rabbit anti-mouse Iba-1 (1:2000, Wako Chemicals, Richmond, VA, USA) or anti-phospho-STAT1 (Tyr701) (58D6, rabbit mAb) (1:500, Cell Signaling Technology, Beverly, MA, USA) primary antibodies, washed thrice with Tris-buffered saline, and incubated with secondary antibodies in UltraMap anti-Rb horseradish peroxidase (HRP) (Ventana Medical Systems, Inc.). Streptavidin-biotin-HRP complex (sABC/HRP) was detected with the ChromoMap DAB detection kit (Ventana Medical System, Inc.). The immunostained sections were then counterstained with hematoxylin (Ventana Medical System, Inc.) and plated on cover slips using Canadian balsam solution (Polysciences Inc., Warrington, PA, USA). Reactions were observed using a light microscope (PM-20; Olympus Corporation) and photomicrographs were acquired with a BX-51 microscope (Olympus Corporation) equipped with a color digital camera (DFC280; Leica Microsystems).

### Levels of Inflammatory and Anti-inflammatory Cytokines in *T. gondii*-Infected Brain Tissue

Cytokine levels of IL-6, IL-12 (p70), IFN-γ, TNF-α, granulocyte-macrophage colony-stimulating factor (GM-CSF), IL-10, IL-4, and TGF-β in *T. gondii*-infected mouse brains were examined using the Bio-Plex mouse cytokine assay kit (Bio-Rad Laboratories, Hercules, CA, USA) and enzyme-linked immunosorbent assay (ELISA) kits (R&D Systems, Inc., Minneapolis, MN, USA). Brain tissues of C57BL/6 mice were obtained at 0, 3, 6, 9, and 12 weeks PI, and lysed using the MicroRotofor Cell Lysis Kit (Bio-Rad Laboratories) (*n* = 3). The soluble homogenate proteins were quantified using a bicinchoninic acid (BCA) assay kit (Pierce Biotechnology, Inc., Rockford, IL, USA). The Bio-Plex assay was performed according to the manufacturer’s instructions and the raw data [mean fluorescent intensities (MFI)] were analyzed with Bio-Plex Manager Software (Bio-Rad Laboratories) to obtain concentration values. The one-way analysis of variance (ANOVA) was used for statistical analysis, and the results were analyzed using Bio-Plex Data Pro™ software. Cytokine analysis was performed using ELISA kits (R&D Systems, Inc.) according to the manufacturer’s protocol. The reaction was measured at 450 nm using an Epoch microplate reader (BioTek Instruments, Inc., Winooski, VT, USA) and cytokine concentrations were calculated using a standard curve of the corresponding cytokine provided with the ELISA kit.

### Microarray Analysis of *T. gondii*-Infected Brain

Total RNA of *T. gondii*-infected brain tissues was separately extracted at 0, 3, 6, 9, and 12 weeks PI, and pooled for microarray analysis (*n* = 3), which was performed by Macrogen Inc. (Seoul, South Korea) using an Illumina MouseRef-8 v2 Expression BeadChip array (Illumina, Inc., San Diego, CA, USA). Briefly, 0.55 µg of total RNA was amplified using the Illumina TotalPrep RNA Amplification Kit (Ambion, Austin, TX, USA) and purified using the Ambion Illumina RNA amplification kit (Ambion) to yield biotinylated cRNA. Following fragmentation, 0.75 µg of cRNA were hybridized to the Mouse Expression BeadChip (Illumina, Inc.) according to the manufacturer’s protocol. Arrays were scanned with the Illumina Bead Array Reader Confocal Scanner. Array data export processing and analysis was performed using Illumina GenomeStudio v2011.1 (Gene Expression Module v1.9.0), and the data were analyzed with R v. 2.15.1 statistical software. Hierarchical cluster analysis was performed using Permute Matrix EN software. All heat maps were generated using Excel Spreadsheet Software (Microsoft Corporation, Redmond, WA, USA) with conditional formatting. The expression rates of each gene at 3, 6, 9 and 12 weeks PI were compared to baseline (week 0 PI). Positive correlations are depicted in yellow (increased expression) and negative correlations (decreased expression) are depicted in blue. Heat maps of inflammatory and anti-inflammatory cytokines, microglia phenotype markers, and immune regulatory markers are represented by color scales. Each row represents cytokines and immune markers, and each column represents infection times from 0 to 12 weeks PI. The color scale of the heat map corresponds the relative minimum (−3) and maximum (+3) values of each cytokine.

### TLAs Prepared from Tachyzoites of *T*. *gondii* Strains RH and ME49

*Toxoplasma gondii* lysate antigens of strain RH or ME49 were prepared as previously described with slight modifications (8, 11). Briefly, peritoneal exudates of infected mice at day 4 PI were passed twice through a 25-gauge needle and then through a 5-µm Millex filter membrane (Merck Millipore, Tullagreen, Ireland) to remove debris and host cells. Then, tachyzoites of *T. gondii* strain RH were recovered and washed with sterilized PBS (pH 7.2). Parasites were then washed and suspended in PBS (pH 7.2) for further antigen preparation. To prepare ME49 tachyzoite antigens, 20 cysts of strain ME49 were intraperitoneally injected to BALB/c mice to obtain ME49 tachyzoites converted from cysts. At 6 to 8 days PI, the tachyzoites were harvested by washing the peritoneal cavity with PBS. Tachyzoites of both *T. gondii* strains RH and ME49 were cultured in Vero cells (monkey kidney cells, KCLB cell line; no. 10081) grown in complete Roswell Park Memorial Institute 1640 media (WelGENE Inc., Daegu, Korea) supplemented with 100 µg/mL of penicillin (Gibco/BRL, Grand Island, NY, USA), 100 µg/mL of streptomycin (Gibco/BRL), and 5% fetal calf serum (Lonza, Walkersville, MD, USA) at 37°C under an atmosphere of 5% CO_2_. After culturing in Vero cells, tachyzoites of strains ME49 and RH were passed through a 25-gauge needle twice, and then debris and cells were removed by passing through 5-µm filter membranes. After washing, the tachyzoites were re-suspended in PBS, sonicated on ice, and then centrifuged. Supernatants, containing the TLA fractions (respectively named as RH-TLA and ME-TLA) were filtered through 0.22-µm filter membranes (Millipore Corp., Bedford, MA, USA). Proteins in the TLA fraction were quantified using a BCA commercial reagent (Pierce Biotechnology, Inc.) and stored at −80°C until used.

### Flow Cytometry Analysis to Determine the Microglia Phenotype of BV-2 Cells after Treatment of the *T*. *gondii* Antigen

Antigens against *T*. *gondii* strain RH or ME49 tachyzoites (40 µg/mL) and/or recombinant IFN-γ (100 ng/mL; PeproTech, Rocky Hill, NJ, USA) were used for *in vitro* activation of BV-2 cells, a murine microglial cell line. BV-2 cells were cultured in Dulbecco’s modified essential medium (Applied Scientific, San Francisco, CA, USA) supplemented with 10% heat-inactivated fetal calf serum (Hyclone, Ogden, UT, USA), 4 mM l-glutamine, 0.2 mM penicillin, 0.05 mM streptomycin, and 20 mM HEPES (Sigma-Aldrich Corporation, St. Louis, MO, USA) at 37°C under an atmosphere of 5% CO_2_ (8). After incubation for 24 h, BV-2 cells were harvested for further fluorescence-activated cell sorting (FACS) analysis. The following anti-mouse antibodies were used for flow cytometry analysis of the cultured BV-2 microglial cells: fluorescein isothiocyanate (FITC)-conjugated anti-CD80 (eBioscience, Inc., San Diego, CA, USA), phycoerythrin (PE)-conjugated anti-CD86 (eBioscience, Inc.), FITC-conjugated anti-CD274 (PD-L1) (B7-H1; eBioscience, Inc.), PE-conjugated anti-CD273 (PD-L2) (B7-DC; eBioscience, Inc.), allophycocyanin (APC)-conjugated anti-major histocompatibility complex (MHC) II (CD74, eBioscience), PE-conjugated anti-CD40 (eBioscience, Inc.), and FITC-conjugated anti-CD206 (BioLegend, San Diego, CA, USA). All staining processes for FACS analysis were conducted in accordance with the manufacturer’s protocols with FACS staining buffer (PBS containing 1% bovine serum albumin and 0.1% sodium azide). Samples were analyzed using a FACSCalibur flow cytometer (BD Immunocytometry Systems, San Jose, CA, USA) with forward/side scatter gates to exclude nonviable cells and the data were analyzed using FlowJo software (Tree Star, Inc., Ashland, OR, USA). Data are presented as the mean (±SD) fluorescence intensity (MFI). In this study, *in vitro* experiment of BV-2 cell culture was data obtained after conducting three individual experiments.

### Western Blot Analysis

Total proteins were extracted from uninfected and infected whole mouse brains using the PRO-PREP™ Protein Extraction Kit (iNtRON Biotechnology, Seongnam-Si, Korea) and quantified with a NanoDrop spectrophotometer (NanoDrop Technologies, Oxfordshire, UK). Proteins were separated by sodium dodecyl sulfate-10% polyacrylamide gel electrophoresis at 100 V for 110 min and then transferred to a nitrocellulose membrane (Bio-Rad Laboratories) using the Mini Trans-Blot^®^ Electrophoretic Transfer Cell (Bio-Rad Laboratories) at 80 V for 100 min. The membranes were then incubated with the primary antibodies goat anti-SOCS1 (ab9870; Abcam, Cambridge, UK) (1:200) and mouse anti-β-actin (sc-47778; dilution, 1:500; Santa Cruz Biotechnology, Dallas, TX, USA), followed by the secondary antibodies donkey anti-goat immunoglobulin (Ig)G-HRP (sc-2020; dilution, 1:2,000; Santa Cruz Biotechnology) and goat anti-mouse IgG-HRP (sc-2005; dilution, 1:4,000; Santa Cruz Biotechnology). Signals were detected by exposing the membrane to chemiluminescence HRP substrate (Thermo Fisher Scientific) using a Fuji LAS1000 Lumino Image Analyzer (Fujifilm Corporation, Tokyo, Japan).

### Nitrite and Urea Production by BV-2 Cells Stimulated by Various Cytokines and Strain-Specific Tachyzoite Antigens (RH-TLA and ME-TLA)

BV-2 cells were incubated for 24 h in 6-well culture plates (SPL Lifesciences Co., Ltd., Pocheon, South Korea) with either 100 ng/mL of IFN-γ (PeproTech), 20 ng/mL of IL-4 (Prospec-Tany Technogene Ltd., Rehovot, Israel), and/or one of the *T. gondii* antigens (RH-TLA or ME-TLA) at a concentration of 20 or 40 µg/mL. Culture supernatants were collected and assayed to determine contents of nitrite and urea, which reflect NO production and Arg1 levels, respectively. NO production was determined using Griess reagent (Sigma-Aldrich Corporation). After 50 µL of culture supernatant was reacted with 50 µL of Griess reagent in each well of a 96-well plate (SPL Lifesciences Co., Ltd.), the reaction was measured using a microplate reader at an optical density (OD) of 540 nm (Biotech, VT, USA). Urea concentration was determined using a commercial urea kit (Abnova Corporation, Taipei, Taiwan) according to the manufacturer’s protocol. In brief, 50 µL of culture supernatant was mixed with 50 µL of distilled water and the solution was reacted with 200 µL of a working reagent for 20 min. Then, the OD value of the sample was measured at 520 nm and the concentrations of nitrite and urea were calculated using standard curves. *In vitro* experiment of BV-2 cell culture was data obtained after conducting three individual experiments.

### mRNA Levels of iNOS, COX-2, and Arg1 in *T. gondii*-Infected BV-2 Cells

BV-2 cells were infected with tachyzoites of *T. gondii* strain RH or ME49 *in vitro* at an effector to target ratio (E:T ratio; *T*. *gondii*:BV-2 cell) of 1:5 for 24 h. Total RNA of the cultured cells was extracted using the RNeasy kit (Qiagen). All samples were reverse transcribed using RT premix (Elpis Biotech Inc.) and the iQ5 real-time PCR detection system (Bio-Rad Laboratories) with SYBR green master mix (Enzynomics, Cheongju, South Korea) under the following condition: initial denaturation at 50°C for 5 min and 95°C for 10 min, followed by 40 amplification cycles of denaturation at 95°C for 10 s and annealing at 60°C for 30 s, with a final extension cycle at 72°C for 5 min. Specific amplification was verified by analysis of the melting curve and separation of the RT-PCR products on a 3% agarose gel. Data analysis was performed using iQ™5 optical system software (Bio-Rad Laboratories). The following primer sequences and amplicon sizes were retrieved from the PrimerBank database^2^: GAPDH (PrimerBank ID 6679937a1) forward 5′-AGG TCG GTG TGA ACG GAT TTG-3′ and reverse 5′-TGT AGA CCA TGT AGT TGA GGT CA-3′ (123 bp); Arg1 (PrimerBank ID7106255a1) forward 5′-CTC CAA GCC AAA GTC CTT AGA G-3′ and reverse 5′-AGG AGC TGT CAT TAG GGA CAT C-3′ (185 bp); Nos2 (iNOS) forward 5′-GTT CTC AGC CCA ACA ATA CAA GA-3′and reverse 5′-GTG GAC GGG TCG ATG TCA C-3′(127 bp); and Ptgs2 (COX-2) forward 5′-TGT GAC TGT ACC CGG ACT GG-3′ and reverse 5′-TGC ACA TTG TAA GTA GGT GGA C-3′(233 bp).

### Statistical Analysis

All statistical analyses were performed using Microsoft Excel and GraphPad Prism 5 software (GraphPad Software, Inc., La Jolla, CA, USA). Data are presented as the mean ± SD. One-way ANOVA followed by the Bonferroni multiple-comparison test were used to assess differences between experimental groups. A probability (*p*) values of <0.05 was considered statistically significant. * indicates significant difference by one-way ANOVA compared with the control. ^#^ indicates significant difference between the experimental groups.

## Results

### Histopathological Changes and Microglia Activation in the Hippocampal DG during the AI to CI Stage of *T. gondii*

**Figure 1:**
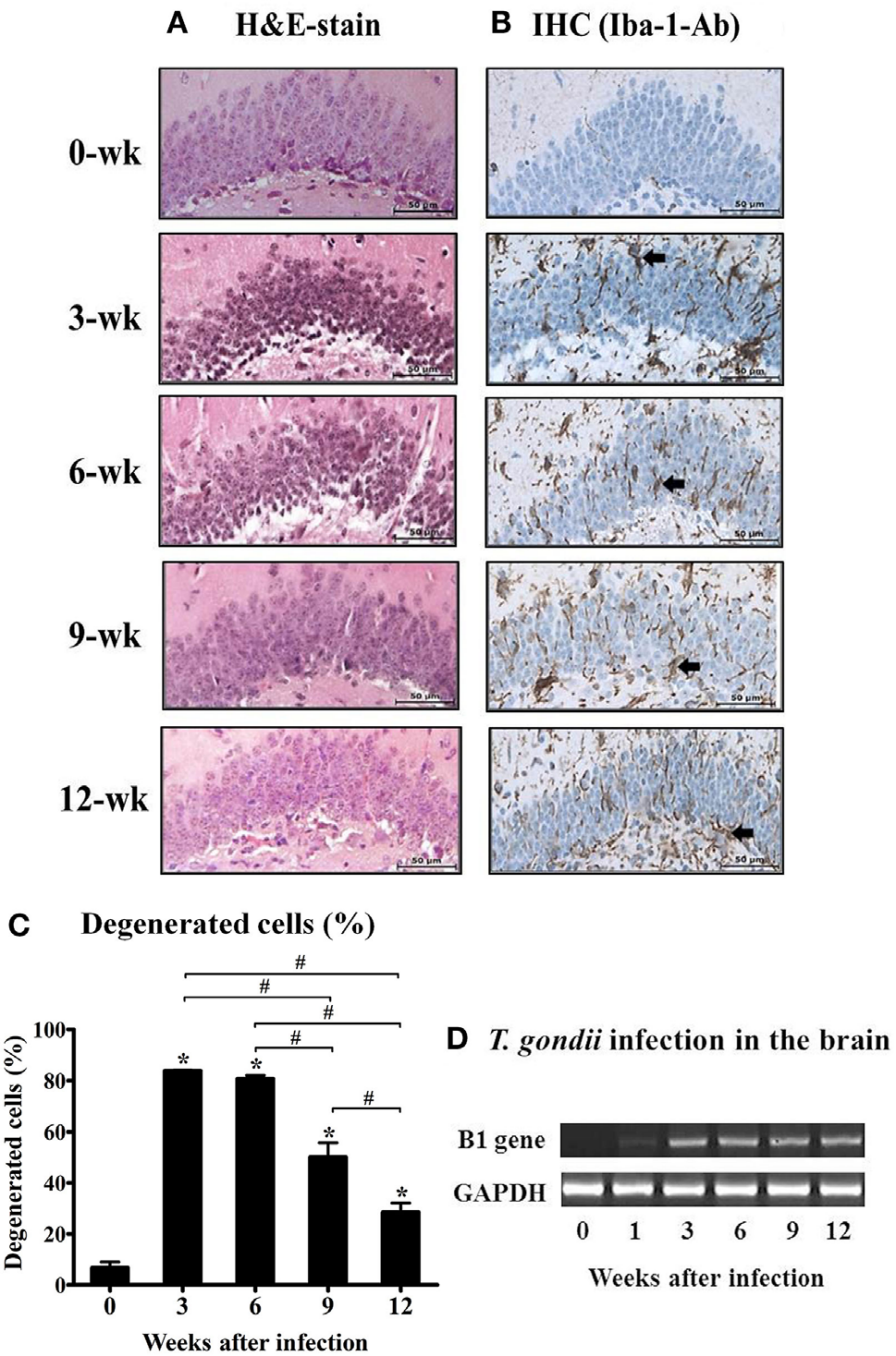
Changes in neuronal degeneration and proliferation of microglial cells in the hippocampal dentate gyrus during *T. gondii* infection. The brain tissues were harvested at 0, 3, 6, 9, and 12 weeks postinfection, and subjected to histological staining. **(A)** hematoxylin and eosin staining, 400×. **(B)** Immunohistochemistry (IHC) results of the brain tissue stained with Iba-1-antibody (brown color), 400×. Scale bar = 50 µm. **(C)** Degenerated cells in the brain tissues after *T*. *gondii* infection were counted and are expressed by percentage (%).* indicates significant difference compared with the control. ^#^ indicates significant difference between the experimental groups. **(D)** *T. gondii*-specific B1 gene expression and RT-PCR products were loaded into 1% agarose gels (98 bp).

To observe the neuronal cell damage caused by *T. gondii* infection, histopathologic changes in the hippocampal region were examined by H&E staining. As shown in Figure 1A, purple-colored normal and un-injured neurons were observed with a light microscopic throughout most of the hippocampal DG (Figure 1A, week 0), whereas *T. gondii* infection resulted in an increase the proportion of eosinophilic neurons, which were characterized by cell body shrinkage, intensely stained eosinophilic cytoplasm, and small/shrunken darkly stained nuclei (Figure 1A, at 3 and 6 weeks PI). The appearance of eosinophilic neurons was increased during the AI stage and then significantly decreased during the CI stage from 6.7 ± 3.88% at week 0 to 83.8 ± 0.57% at week 3, 80.8 ± 2.38% at week 6, 50.1 ± 9.66 at week 9, and 28.5 ± 6.13% at week 12 PI (Figure 1C, *p* < 0.05). At this time, activation and infiltration of microglial cells in the DG were evaluated by immunohistochemical analysis with antibody against Iba-1, an activation marker of microglia (Figure 1B). The infiltration of Iba-1-positive cells (colored in brown) during hippocampal formation had increased from 3 weeks PI and was sustained during the 12-week experimental period (Figure 1B). These histopathological changes appeared after infection of *T. gondii*, which were detected by monitoring of the B1 gene (Figure 1D). Importantly, this result suggests that neurodegeneration increases during the AI stage and decreases in the CI stage (9 weeks PI), despite the presence of harmful signs in the brain tissue, such as activation of inflammatory microglia and continuity of *T. gondii* infection. Accordingly, further analysis showed that neurodegeneration accompanying *T. gondii* infection had decreased during the CI stage.

### Profiles of Inflammatory and Anti-inflammatory Cytokines in the Mouse Brain According to the Time of *T. gondii* Infection

**Figure 2:**
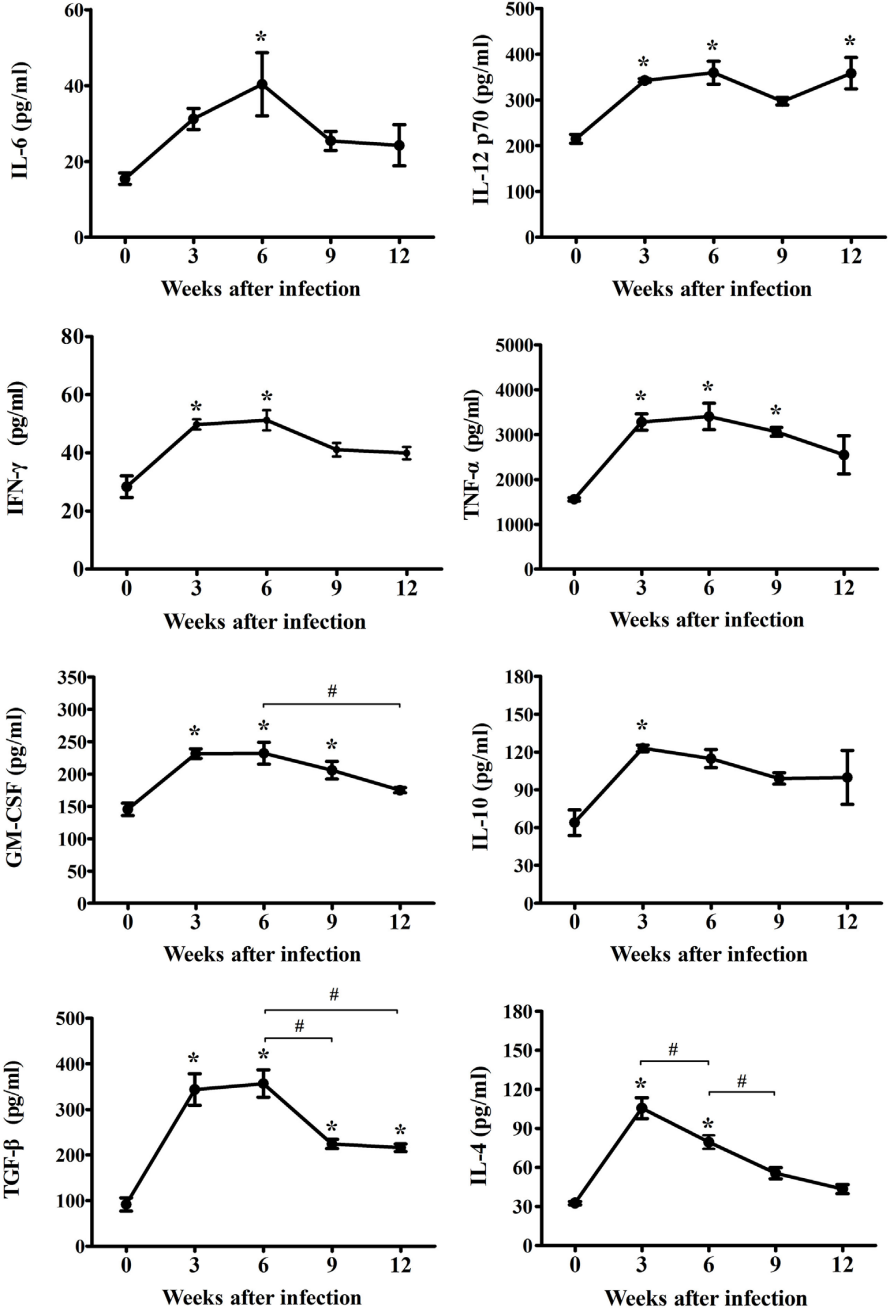
Expression changes of cytokines in cerebral *Toxoplasma gondii* infection from the acute infection stage to the chronic infection stage. Brain tissues were harvested at 0, 3, 6, 9, and 12 weeks postinfection and analyzed for changes in cytokine levels due to infection. Levels of inflammatory cytokines [IL-6, IL-12p70, IFN-γ, tumor necrosis factor (TNF)-α, and granulocyte-macrophage colony-stimulating factor (GM-CSF)] and anti-inflammatory cytokines [IL-4, IL-10, and transforming growth factor-β (TGF-β)]. Cytokine levels are presented as the mean ± SD at each infection stage. *p < 0.05 (one-way analysis of variance). * indicates significant difference compared with the control. ^#^ indicates significant difference between the experimental groups.

To reveal the characteristics of the inflammatory mechanisms underlying microglia activation after *T. gondii* infection, inflammatory, and anti-inflammatory cytokines (IL-6, IL-12p70, IFN-γ, TNF-α, GM-CSF, IL-4, IL-10, and TGF-β) were examined at 0, 3, 6, 9, and 12 weeks PI. As show in Figure 2, the concentrations of inflammatory- (IL-6, IL-12p70, IFN-γ, TNF-α, and GM-CSF) and anti-inflammatory-cytokines (IL-4, IL-10, and TGF-β) had gradually and statistically increased during the AI stage (3–6 weeks PI, * *p* < 0.05 at every time point compared, as compared to week 0). Furthermore, the levels of inflammatory cytokines (IL-6, IL-12p70, IFN-γ, TNF-α, and GM-CSF) had peaked at 6 weeks PI, while those of the anti-inflammatory cytokines (IL-4 and IL-10) had peaked at 3 weeks PI, suggesting that levels of the anti-inflammatory cytokines had decreased immediately after the rapid increase during the early stage of infection (Figure 2). At this time, TGF-β, which is known to be associated with neuroprotection, continued to increase until 6 weeks PI. The peak level of each cytokine is shown in Figure 2. The abundance of the inflammatory cytokines IL-6, IL-12p70, IFN-γ, TNF-α, and GM-CSF had increased from baseline (week 0) to 6 weeks PI by 262, 167, 180, 218, and 160%, respectively, whereas the abundance of the anti-inflammatory cytokines IL-4 and IL-10 had increased by 324 and 192% at 3-weeks PI, respectively, and that of TGF-β had increased by 389% at 6 weeks PI. The cytokine IFN-γ, which produces a noxious cellular effect on the CNS and simultaneously activates the inflammatory cellular responses, had decreased at 6 weeks PI, whereas the levels of IL-12, which activates microglia, were sustained with no remarkable decrease during the experimental period. Moreover, levels of the cytokine TGF-β, which induces neuroprotective anti-inflammatory responses, were mostly sustained during the experimental period, but had decreased slightly. The levels of the anti-inflammatory cytokines IL-4 and IL-10 had decreased significantly during the CI stage (9–12 weeks). The results of the present study showed that levels of the inflammatory cytokines had continually increased to 6 weeks PI and were maintain at high levels even during the CI stage, although there were slight decreases, whereas levels of the anti-inflammatory cytokines had remarkably increased during the AI stage (3 weeks PI) and then decreased immediately thereafter during the CI stage. The most important finding was the significant increase and maintenance of IL-12 and TGF-β levels during the CI stage for 12 weeks PI (Figure 2, * *p* < 0.05).

### Relative Cytokine mRNA Levels and Microglia Phenotype Markers for *T. gondii*-Infected Mouse Brain

**Figure 3:**
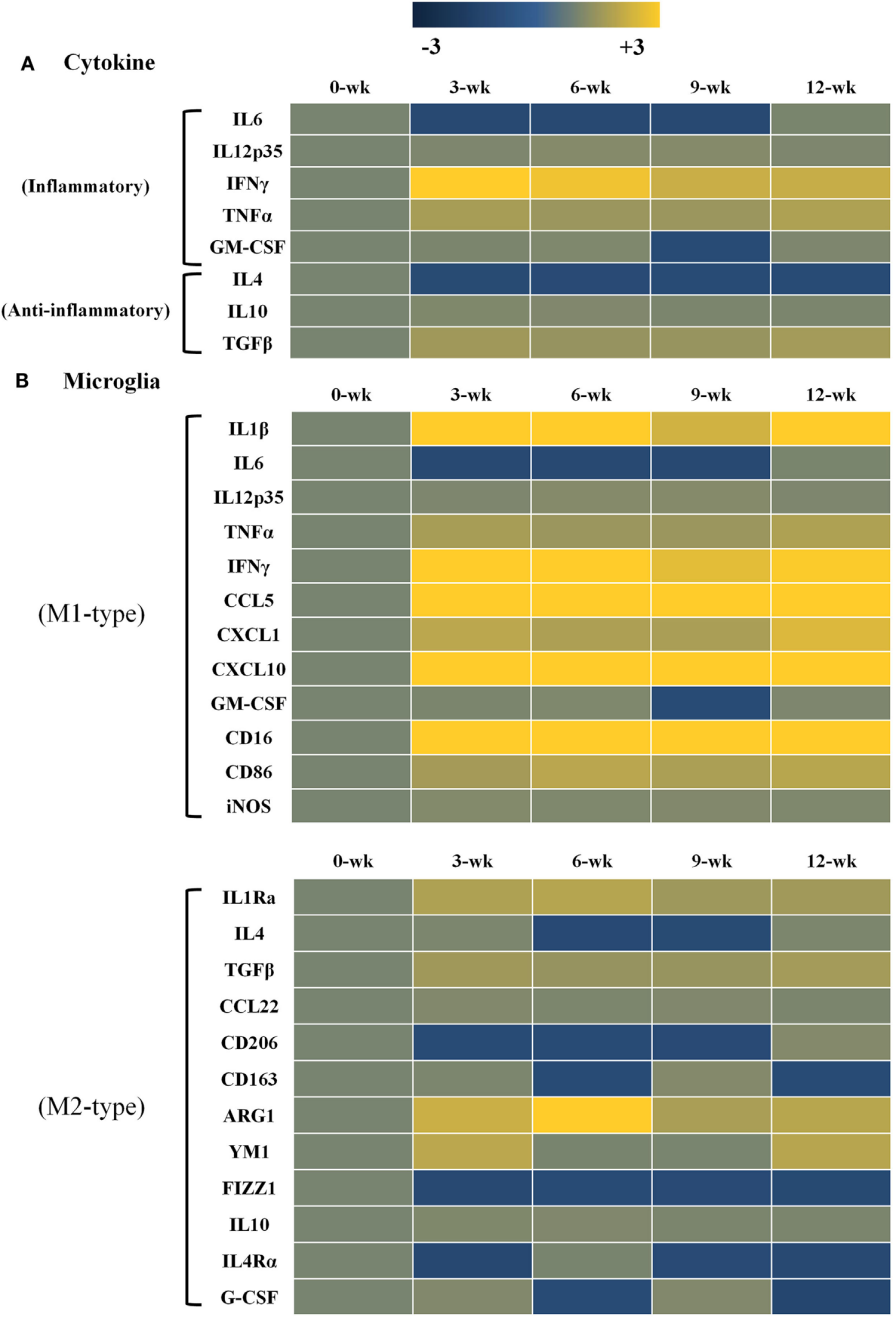
Heat maps representing cytokine and chemokine concentrations in the inflammatory immune response and microglia phenotypes. Transcript levels of inflammatory and anti-inflammatory cytokines as well as microglia phenotype markers were measured in *Toxoplasma gondii*-infected mouse brains using microarray analysis. Each row of the heat map represents cytokines related with inflammatory and anti-inflammatory responses **(A)** as well as microglia phenotype markers related with the M1-type and M2-type **(B)**. Each column represents infection times from week 0 to 12 postinfection. The color scale corresponds to the relative expression of the cytokine for the minimum (−3) and maximum (+3) of all values.

Changes in gene expression levels of inflammatory and anti-inflammatory cytokines after *T. gondii* infection are shown in Figure 3. Gene expression levels of each cytokine at each time point of infection were compared to those of normal brain tissues (onefold at week 0 PI, a faint khaki color). Data are presented as heat maps (Figure 3), which depict the most commonly up- and downregulated transcripts from microarray analysis. The data presented in blue and yellow indicate reduced and increased cytokine expression levels. The present study analyzed gene expression patterns of inflammatory and anti-inflammatory cytokines (Figure 3A), as well as the microglia phenotype markers of the M1 and M2 types (Figure 3B). As compared with the anti-inflammatory cytokines (IL-4, IL-10 and TGF-β; 1.0- to 1.6-fold), transcript levels of the inflammatory cytokines (IL-12, IFN-γ, and TNF-α) had increased by 1.5- to 3.3-fold, as compared to log_2_-values (onefold) of the transcript levels of control mice. Especially, IFN-γ was remarkably increased by 3.3-fold at 3 weeks PI and was constantly maintained at 2.2-fold at 12 weeks PI. Likewise, the inflammatory cytokine TNF-α and the anti-inflammatory cytokine TGF-β were relatively increased by 1.5- to 1.8-fold during the 12-week experimental period. Expression levels of the phenotype markers M1-type and M2-type were found to be polarized in the M1-type microglia (Figure 3B). Most importantly, Arg1 levels among M2-markers were consistently increased for 12 weeks PI at 1.7- to 3.4-fold, even though the increase in IFN-γ levels was 2.2- to 3.3-fold. Further examining of the 12 polarization markers in each type of microglial cell phenotype showed that nine markers of the M1-type (IL-1β, IL-12, TNF-α, IFN-γ, CCL5, CXCL1, CXCL10, CD16, and CD86) and four markers of the M2-type (IL-1Rα, TGF-β, Arg1, and YM1) were consistently expressed for the entire 12-week period after infection (Figure 3B). Most notably, there was no increase in iNOS, while Arg1 was remarkably increased even if polarization and activation of M1-type microglia were distinct during the CI stage (Figure 3B).

### Kinetics of Microglia Activation and Phosphorylation of Stat1 in *T. gondii*-Infected Mouse Brain Tissues

**Figure 4:**
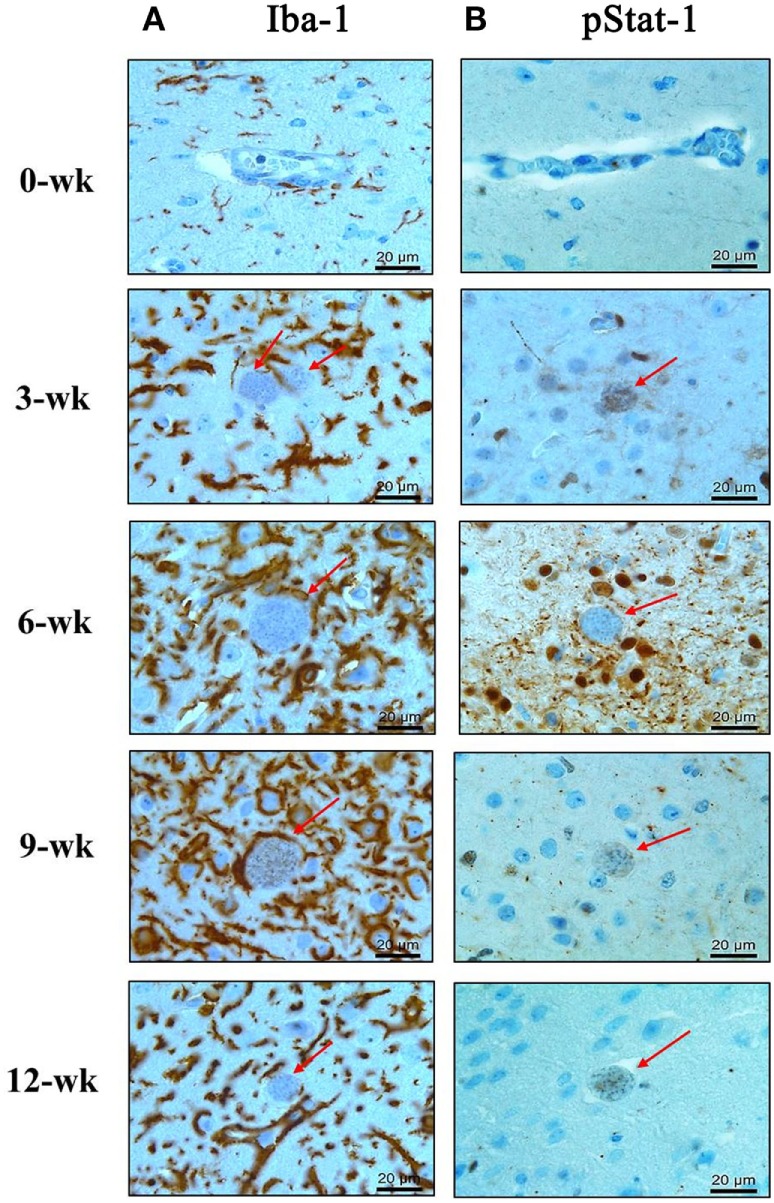
Phosphorylation of Stat1 (pStat1) and Iba-1-stained microglia infiltrated and activated around *Toxoplasma gondii* cysts. *T. gondii*-infected mouse brains were harvested at 0, 3, 6, 9, and 12 weeks postinfection, embedded in paraffin, and immunostained with Iba-1- **(A)** and phosphorylated Stat-1 antibodies **(B)**. *T. gondii* cysts (arrow). Activated microglia [**(A)** brown color]. pStat1 immunoreactivity [**(B)** brown color]. Magnification, 400×. Scale bar = 20 µm.

The above results showed that the expression levels of the inflammatory cytokines (IL-12, IFN-γ, and TNF-α) had continuously increased during the CI stage. Among these cytokines, mRNA and protein levels of IL-12, a key cytokine of microglial cells activation, and IFN-γ, a key cytokine of Th1-helper T-cell activation, were maintained steadily with high levels during the CI stage (Figures 2 and 3). To comprehensively link changes between gene and protein expression levels, brain tissues were immunohistologically stained with Iba-1- and phosphorylated STAT-1 (pStat1) antibodies for microglial activation and to determine whether these molecules participate in the IFN-γ-mediated pathway, respectively (Figures 4A,B). Because IFN-γ plays a role in adaptive cellular immunity against *T. gondii* infection and STAT-1 is important for intracellular downregulation of IFN-γ, the increase in pStat1-stained cells in the brain indicates an increase in IFN-γ-mediated Th1-T-cell immune responses. However, pStat1-stained cells were observed around the cyst (red arrow) over a relatively short period of time at 6 weeks PI and, thereafter, were undetectably in the brain tissues (Figure 4B). At this time, the proportion of microglial cells had remarkably increased around the *T. gondii* cysts (red arrow) during the AI stage and was activated continuously during the CI stage (Figure 4A). This activation of microglial cells can be explained by morphological characteristics, such as hyper-ramification and long, thin processes extending from the cell body into the surrounding milieu (Figure 4A, colored in brown). The increase in activated microglia was greatest at 6–9 weeks PI and then had decreased slightly at 12 weeks PI (CI stage). These results strongly suggest microglia are activated throughout the infection period, whereas STAT1 phosphorylation was limited and no longer maintained during the CI stage.

### Effects of TLA (RH- or ME-TLA) on the Polarization and Activation of Microglial Cells

**Figure 5:**
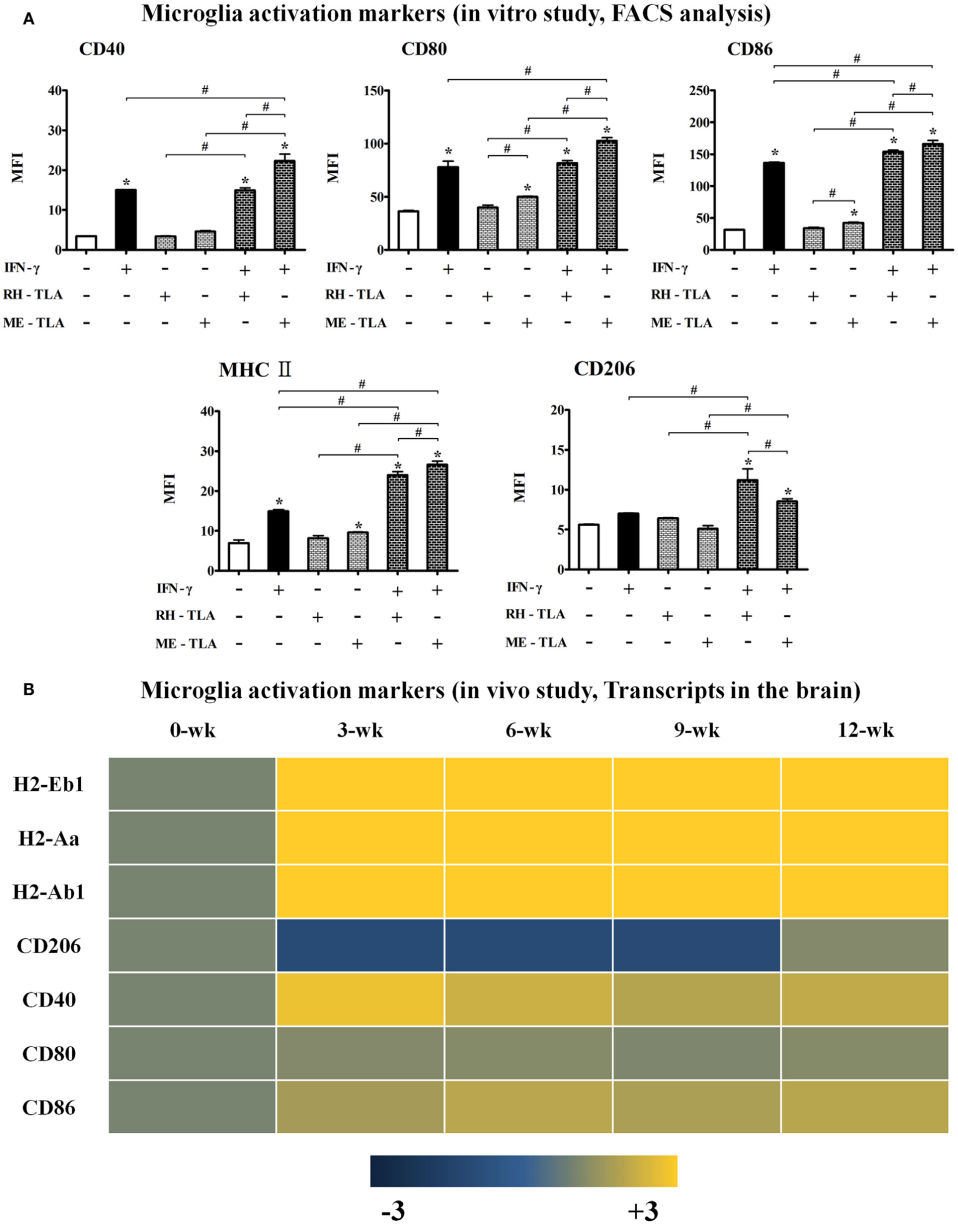
Microglia activation in *Toxoplasma gondii*-infected brain and BV-2 cells stimulated with *T. gondii* lysate antigens (TLAs) (RH-TLA and ME-TLA). Expression levels of major histocompatibility complex II antigens (H2-Eb1, H2-Aa, and H2-Ab1), CD40, and CD86 by FACS analysis. **(A)** Heat map expression of cell surface markers related with microglia activation in *T. gondii*-infected brains, **(B)** results are expressed as the mean ± SD of the mean fluorescence intensity. *p < 0.05 (one-way analysis of variance). * indicates significant difference compared with the control. ^#^ indicates significant difference between the experimental groups.

Infection immunity in *T. gondii*-infected brain tissues can be explained by the immune characteristics regulated by the host–parasite relationship. Infectious immunity shown in the present study was characterized by an increase in microglial cells and the limited IFN-γ-mediated immune response. However, it remains unclear whether these immune responses were induced by immune regulation *via* the host–parasite relationship or by immunological triggering of *T. gondii* antigens as a PAMP. For this purpose, TLAs were prepared from RH- or ME49 tachyzoites to investigate the effects of TLA on the activation and polarization of microglia, which were further compared with transcript levels in the brain (Figures 5A,B). TLAs contain many PAMPs and stimulate toll-like receptor (TLR)-based immune responses. Moreover, because *T. gondii* strains (RH and ME49) have different modes of infectious immunity, the comparison of *T. gondii* antigens (RH-TLA or ME-TLA) on microglia activation is important to evaluate difference in immune responses between the AI and CI stages. As shown by the results, the MFI (FACS analysis) of the activation markers of BV-2 microglial cells [i.e., major histocompatibility complex II (MHCII), CD40, CD80, and CD86] had increased in response to IFN-γ stimulation (Figure 5A). Such an increase in MFI indicates an increase in target molecules in FACS. IFN-γ stimulation of BV-2 cells had increased the expression levels of these activation markers, which was accelerated by the presence of TLA (Figure 5A). At this time, ME-TLA had a greater effect on the increase in activation markers than RH-TLA. In contrast, expression of the M2 polarization marker CD206 was lower in ME-TLA than RH-TLA (Figure 5A). These results are consistent with the microarray results (Figure 5B). Subunit transcripts of MHC class II molecules (H2-Eb1, -Aa, and -Ab1), *Cd40, Cd80*, and *Cd86* were increased (16). In contrast, *Cd206* was decreased, as compared to the control (khaki) (Figure 5B). The M1 markers *Cd40, H2-EB1 (-Aa, -Ab1)* (MHCII), and *Cd86* (B7-2) were upregulated in activated microglial cell and acted as co-stimulatory molecules for further T- and B-cell immune responses. Both mRNA and protein levels of the representative M2 marker CD206 were decreased simultaneously in cells infected with *T. gondii* strain ME49, as well as by antigen treatment (ME-TLA) as PAMPs. Accordingly, our results showed that both *TLA* treatment and *T. gondii* infection induced the activation and M1 polarization of microglial cells compared with the control (no-treatment of IFN-γ and lysate antigens *in vitro* study as well as 0-week PI *in vivo* study).

### Changes in Transcripts for T-Cell Differentiation- and T-Cell Exhaustion-Markers in the Brain during *T. gondii* Infection

**Figure 6:**
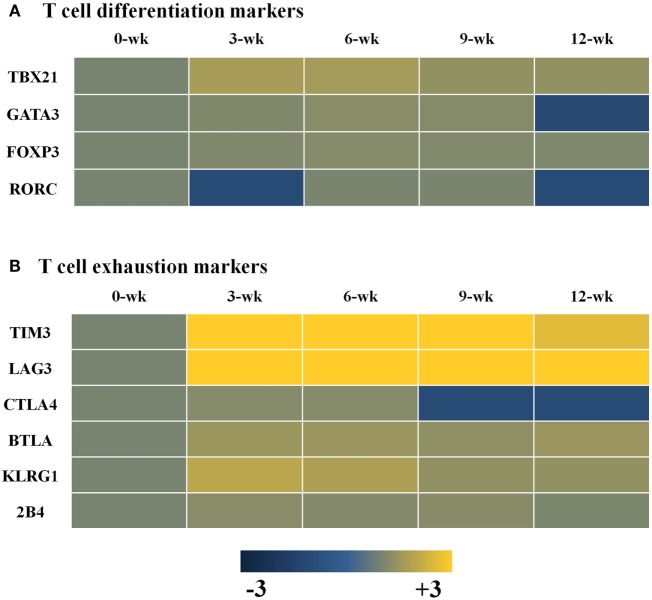
Changes in T-cell differentiation **(A)** and exhaustion **(B)** markers in *Toxoplasma gondii*-infected brain tissues. Expression values represent the intensity of gene expression varying from −3 to +3 colored with blue or yellow.

CI of *T. gondii* in the brain may induce T-cell exhaustion and dysfunction. The results of this study showed a decrease in the IFN-γ-mediated immune response during CI even if microglial cell activation was maintained. To arrive at a possible explanation, markers of T-cell differentiation and dysfunction were examined, which showed that Th1 cell-specific transcription factor TBX21 that controls the expression of IFN-γ was mainly increased during CI, as compared to the Th2 cell-secreted cytokines *Gata3* and *Foxp3*, which promote the functions of regulatory T cells (Figure 6A). Nevertheless, markers of T-cell exhaustion, including *Tim3* and *Lag3*, were also increased simultaneously from the AI stage at 3 weeks PI (Figure 6B). *Tim3* and *Lag3* are known to negatively regulate T-cell proliferation, homeostasis, and Th1 immunity, and act as immune checkpoints. This result means that *T. gondii* infection actively influences the induction of the host immune response against infection. In other words, although these findings were limited to transcript profiles, *T. gondii* infection of the brain simultaneously induces both T-cell differentiation and exhaustion from the AI stage at 3 weeks PI, and maintains the immune environment during the entire the CI stage (Figure 6).

### Changes in mRNA Levels of Immune Effector and Checkpoint Molecules in *T. gondii*-Infected Brain Tissues

**Figure 7:**
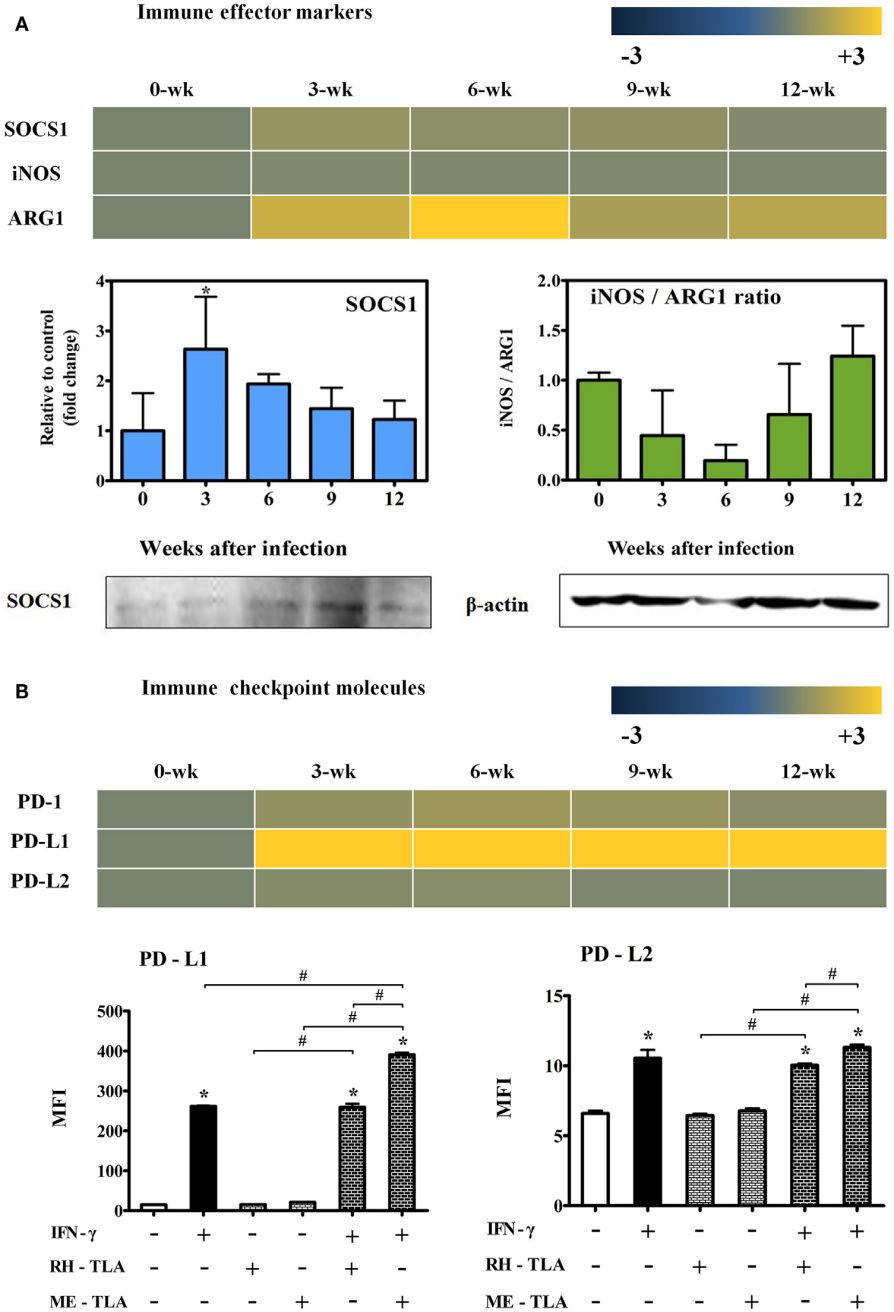
Changes in immune effector and checkpoint molecules. Transcripts of immune effector markers [nitric oxide synthase (iNos) and Arg1] **(A)**, immune control marker (Socs1) **(A)** and immune checkpoint markers (PD-1, PD-L1 and PD-L2) **(B)**. RT-PCR results (SOCS1 and the iNos/Arg1 ratio) and western blot (SOCS1 and β-actin) **(A)**. Transcript expressions(PD-1, PD-L1, and PD-L2) and FACS analysis (PD-L1 and PD-L2) **(B)**. Results are expressed as the mean ± SD of the mean fluorescence intensity (MFI). *p < 0.05 (one-way analysis of variance). * indicates significant difference compared with the control. ^#^ indicates significant difference between the experimental groups.

To investigate the expression of effector molecules associated with both inflammatory and anti-inflammatory responses, changes in transcripts and quantitative gene levels of Socs1, iNos, and Arg1 during the 12-week experimental period were compared to those at baseline (week 0 PI) (Figure 7A). Transcript levels of Socs1, which is thought to be an important immune regulatory factor in this study, was increased by 1.5-fold at 3-weeks PI and maintained during the CI stage (Figure 7A). Likewise, quantitative gene expression levels of Socs1 was the highest at 3 weeks PI (2.63-fold) with statistical significance and, thereafter, had slightly decreased during the CI stage. Similarly, the results of western blot analysis showed an increase in SOCS1 expression during the infection period. In contrast, there was no significant increase in iNOS mRNA levels, and quantitative gene expression levels of iNOS/Arg1 were decreased even during the AI stage (3 and 6 weeks PI). This result is also supported by transcript level of Arg1 (2.2- and 3.4-fold at 3 and 6 weeks PI, respectively) (Figure 7A). Expression levels of the of the immune inhibitory checkpoint molecules PD-1, PD-L1, and PD-L2, as related with T-cell dysfunction, were examined at the transcript level and by FACS analysis. As shown in Figure 7B, transcript levels of PD-1, an inhibitory receptor of T cells, were slightly increased by 1.1- to 1.4-fold at week 12 PI, whereas levels of PD-L1 were remarkably increased by 19.5-, 21.4-, 18.9-, and 23.0-fold at 3, 6, 9, and 12 weeks PI, respectively. This result was also strongly supported by the FACS results expressed as the MFI, suggesting strong activation of the M1-type microglia. When RH- or ME-TLA as PAMPs of *T. gondii* stimulate BV-2 microglial cells, the increase in the MFI of PD-L1 was greater than that of PD-L2 and the degree of increase was greater *via* stimulation of ME-TLA than RH-TLA. Accordingly, it is expected that the immune-triggering effect of *T. gondii* strain ME49 during CI seems to limit the expression of inflammatory effector molecules such as iNOS, even with activation of innate immunity by microglial cells.

### Effects of *T. gondii* Antigens (RH- and ME-TLA) on Nitrite and Urea Production according to Microglia Polarization

**Figure 8:**
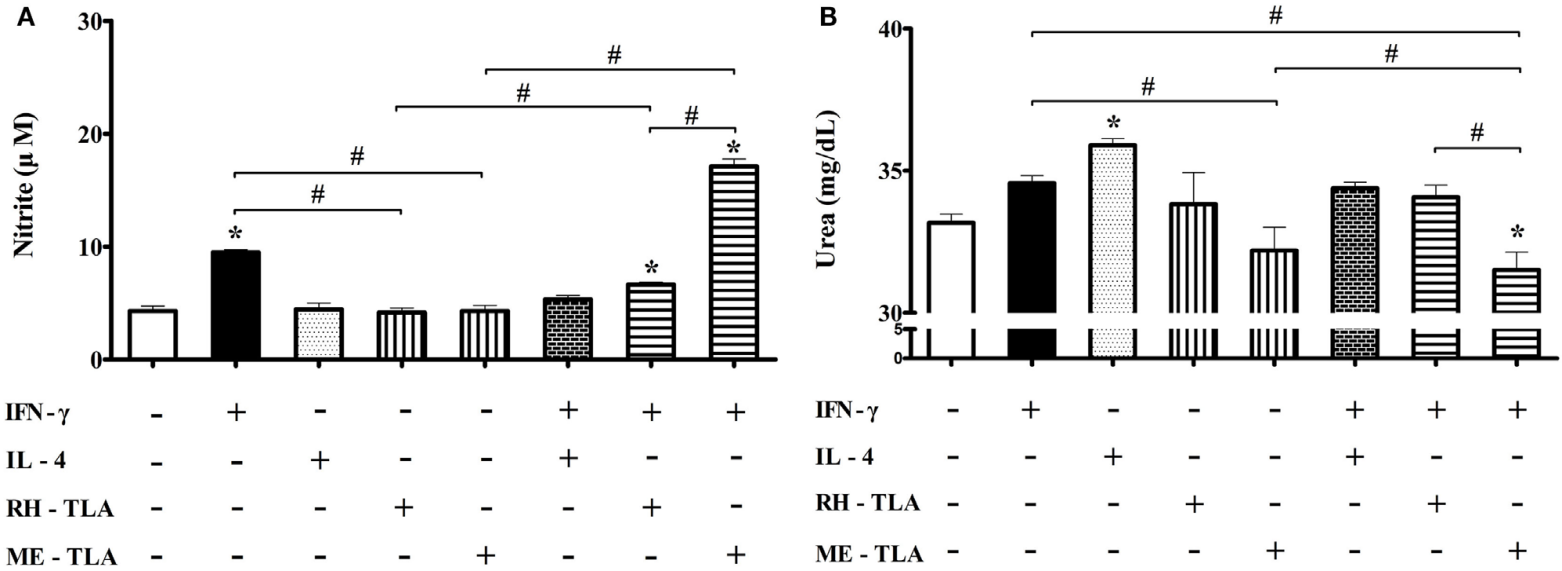
Nitrite and urea production in BV-2 microglia stimulated with recombinant cytokines affecting microglia polarization (IFN-γ and IL-4) or *Toxoplasma gondii* antigens (RH-TLA or ME-TLA). Nitrite (μM) **(A)** and urea (mg/dL) concentrations **(B)**. Data are presented as the mean ± SD. *p < 0.05 (one-way analysis of variance). * indicates significant difference compared with the control. ^#^ indicates significant difference between the experimental groups.

**Figure 9:**
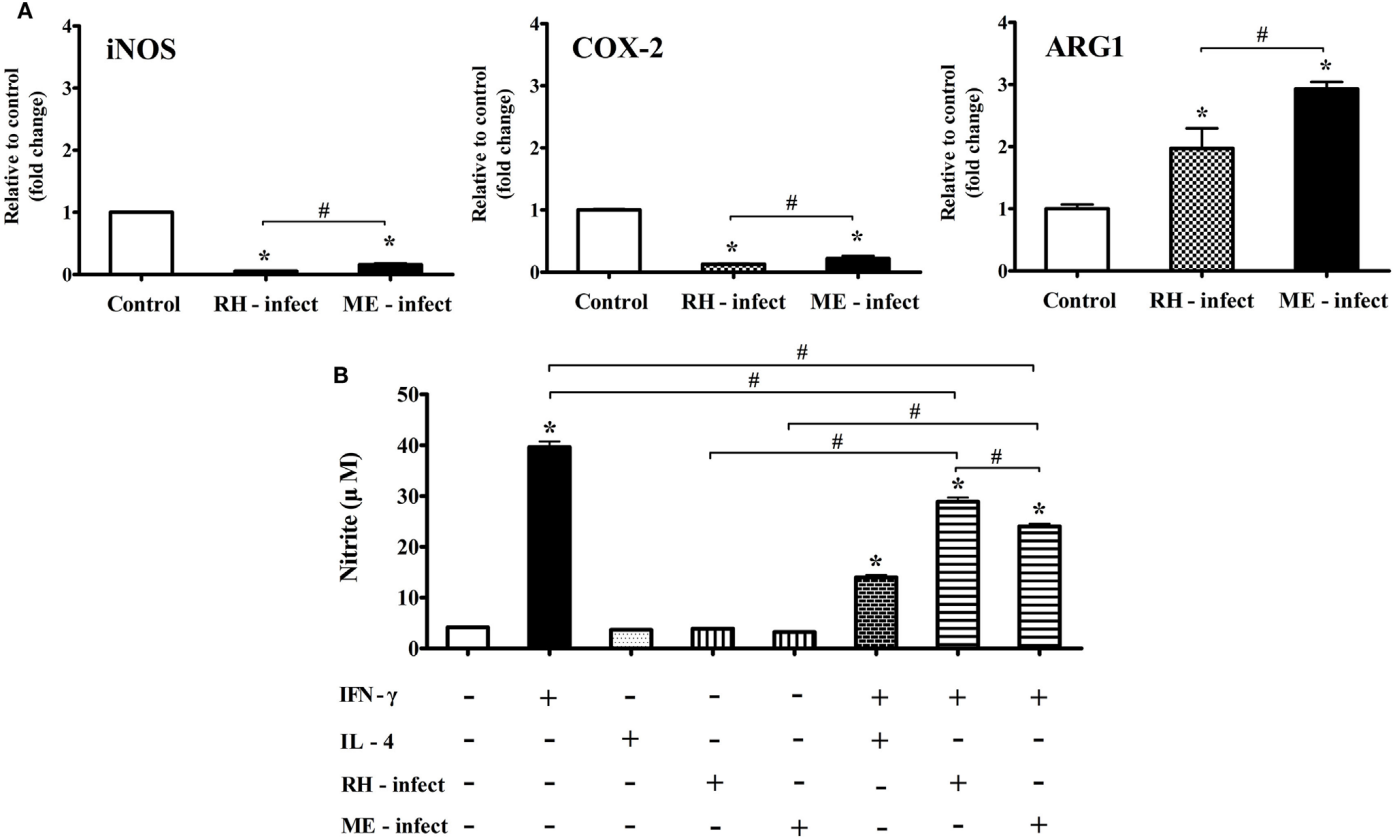
mRNA levels of nitric oxide synthase (iNos), Cox-2, and Arg1, and nitrite production in *Toxoplasma gondii*-infected BV-2 microglial cells. **(A)** RT-PCR analysis of iNos, Cox-2, and Arg1 in *T. gondii* tachyzoites (strains RH and ME49)-infected BV-2 cells. **(B)** Nitrite concentration (μM) in culture supernatant treated with recombinant IFN-γ and/or IL-4, and/or *T. gondii* tachyzoites. Data are presented as the mean ± SD. *p < 0.05 (one-way analysis of variance). * indicates significant difference compared with the control. ^#^ indicates significant difference between the experimental groups.

For 12 weeks PI, iNOS production was decreased and Arg1 production was increased. As well, when BV-2 cells were treated with *T. gondii* antigens as a PAMPs, expression levels of the activation markers were increased. In this regard, an aim of this study was to investigate whether treatment of BV-2 microglial cells with *T. gondii* antigens (RH-TLA or ME-TLA) can induce changes in NO and urea production, as with *T. gondii*-infected brain tissues. In general, nitrite production is mainly increased as a result of M1-type activation, whereas urea production is increased by M2-type microglia activation. The results of the present study showed that the production of nitrite was increased by stimulation of IFN-γ alone (9.5 ± 0.3 µM) or IFN-γ with *T. gondii* antigen (6.6 ± 0.2 µM with RH-TLA and 17.1 ± 0.7 µM with ME-TLA), as compared to the control (4.3 ± 0.4 µM) (Figure 8A). At this time, nitrite concentration had significantly increased *via* stimulation with ME-TLA, as compared to RH-TLA, suggesting that ME-TLA itself is more preferable for the induction of M1-type microglial activation (*p < 0.05). In contrast, urea concentration was increased significantly by stimulation of IL-4, as compared to the control (36.0 ± 0.2 vs. 33.2 ± 0.3, respectively) (Figure 8B). As well, urea production was more decreased by ME-TLA than RH-TLA (32.2 ± 0.8 vs. 33.8 ± 1.1 mg/dL, respectively). Notably, treatment with IFN-γ + ME-TLA had decreased urea production even further than with treatment of IFN-γ + RH-TLA (31.5 ± 0.6 and 34.1 ± 0.4, respectively, *p < 0.05). These results clearly show that treatment of BV-2 cells with ME-TLA induced M1 polarization and M1-activation, which subsequently remarkably increased nitrite production and decreased urea production, suggesting that the immune induction of ME-TLA acts as a PAMP. However, this result is in contrast to the transcript results of the *T. gondii*-infected brain tissues. To clarify this difference, infection of BV-2 cells was performed *in vitro* (Figure 9).

### Effects of *In Vitro T. gondii* Infection (RH- and ME-Strain) on mRNA Expression (iNOS, Cox-2, and Arg1) and Nitrite Production

To elucidate the differences in inflammatory responses and NO production between *T. gondii* infection in the brain and *in vitro* experiments with the *T. gondii* antigens (RH-TLA and ME-TLA), BV-2 cells were infected with *T. gondii* tachyzoites *in vitro*. As shown in Figure 9, the expression levels of the inflammatory response markers iNos and Cox-2 were significantly decreased after *T. gondii* infection *in vitro*, whereas that of Arg1 was significantly increased. Notably, Arg1 expression levels at 24 h after *in vitro* RH and ME49 infection were increased by 2.0 ± 0.3- and 2.9 ± 0.1-fold, respectively, as compared to control (*p < 0.05). Likewise, Arg1 induction was significantly greater after infection with strain ME49, as compared to strain RH. At this time, nitrite production was the highest with treatment of IFN-γ alone (39.6 ± 2.0 µM) and decreased significantly with IFN-γ + *T. gondii* infection (RH and ME49) (28.9 ± 1.4 and 24.0 ± 0.9 µM, respectively, *p < 0.05). As compared to IFN-γ alone, nitrite levels were 27% lower with IFN-γ + *T. gondii* strain RH infection and 39.4% lower with IFN-γ + *T. gondii* strain ME49 infection. Accordingly, these results emphasize that *in vitro T. gondii* infection induces polarization of microglial cells into the M1-type. However, simultaneous inhibition of harmful inflammatory factors, such as NO, had increased Arg1 expression for further immune regulation. This is an important result showing that *in vivo* and *in vitro* infection was consistent. In other words, TLAs acting as PAMPs had strongly induced polarization of M1-type microglial cells and inflammatory responses, while infection of *T. gondii* (*in vivo* and *in vitro*) induced polarization of M1-type microglial cells. However, it simultaneously inhibited a detrimental inflammatory response, such as NO production, *via* Arg1 induction. This host–parasite relationship seems to be a strategy of *T. gondii* to maintain a CI in the brain.

## Discussion

The results of our previous study demonstrated that *T. gondii* infection decreased the neurodegeneration in a Tg2576 mouse model of Alzheimer’s disease (8). In that study, *T. gondii* infection of the brain inhibited neuronal degeneration, as well as learning and memory impairments in Tg2576 mice. As a possible reason, we proposed that the increase in the expression levels of the anti-inflammatory cytokines IL-10 and TGF-β, as well as decreased NO production had favorable effects of *T. gondii* infection and the pathogenesis and progression of Alzheimer’s disease in mice (8). However, because inflammatory responses induced by IFN-γ and NO are essential for the control of *T. gondii* infection, NO regulation in the CNS is very important to control both parasitic proliferation and damage to the host tissues. As an extension of the above results, the aim of the present study was to address the progression of infection immunity to identify the appropriate timing to induce an anti-inflammatory response during CI. In particular, because *T. gondii* is a pathogen that can lead to CI of the host, as well as induce encephalitis and neurodegeneration, it is very important to understand the infection immunity related with microglia polarization and harmful inflammatory responses.

Encephalitis is an important symptom of *T. gondii* infection, although the neuronal degeneration associated with neuroinflammation is not a common finding because immune modulation by *T. gondii* infection has been the focus of numerous previous studies (1–5, 8, 9). In the present study, neuronal cell death caused by *T. gondii* infection in the DG region of the hippocampus was increased in the AI stage at 3–6 weeks PI, which then decreased in the CI stage accompanied with slight decreases in the expression levels of the inflammatory cytokines IL-6, IFN-γ, TNF-α, and GM-CSF. Even in this case, the Iba-1 staining intensity of microglial cells was not significantly decreased during the entire 12-week experimental period. In fact, the microarray results of the *T. gondii*-infected brain and FACS analysis of *in vitro* cultured BV-2 microglial cells with lysate antigens (RH-TLA and ME-TLA) prepared from tachyzoites of *T. gondii* strains RH and ME49, showed M1 polarization of microglial cells both *in vivo* and *in vitro*. However, high levels of the inflammatory and anti-inflammatory cytokines remained during the 12-week experimental, even though the levels had slightly decreased from 6 weeks PI. Thus, it is necessary to consider why neuronal cell death was highest at 3 weeks PI and then decreased. Clearly, *T. gondii* infection induces a mixed immune response characterized by an increase in the expression levels of inflammatory cytokines and the activation and proliferation of microglial cells, as well as increases in the expression levels of the anti-inflammatory cytokines and Arg1 and decreased expression of iNOS and COX-2 accompanied with NO production.

The strategy of *T. gondii* intracellular parasitism involves the manipulation of an excessive response of immune cells and alterations in macrophage phenotypes to suppress inflammatory response and reduce damage to the host. According to the results of an earlier study, *T. gondii* infection is generally controlled by a strong Th1-type immune response *via* the cytokines IL-12p70, TNF-α, and IFN-γ. However, the recruited monocyte population is converted to an anti-inflammatory phenotype in order to restrain excessive immune responses during infection of *T. gondii* strain RH (3–6, 12, 14, 21, 22). On the other hand, the time of infection for the change in microglial cells and inflammatory responses as a cause of CI of the brain infected with strain ME49 it is not well defined. Some studies have provided important clues to prevent excessive activation of macrophages after *T. gondii* infection (3, 14). For example, *Toxoplasma* rhoptry and dense granule proteins play roles in the modulation of pro-inflammatory responses through the regulation in IFN-γ and NF-kB by GRA15 in addition to STAT3/6 activation by ROP16 (3, 14). ROP16 induces STAT1 tyrosine phosphorylation as well as *Socs1 via* STAT3 or 6 (23). As a result, the authors insisted that the infected hosts are able to induce a proper resistance against *T. gondii* infection as well as protection against excessive inflammatory immunity (3, 14, 23). However, these studies were limited by the failure to explain the promotion of immune modulation during *T. gondii* infection because the experiments employed Raw264.7, J447, DC2.4, or human foreskin fibroblast cells for *in vitro T. gondii* infection and *T. gondii*-infected peritoneal exudate cells for *in vivo* infection (3, 14). So, these studies do not reflect the entire infection process. On the contrary, the present study addressed the changes in the infection immunity during the CI stage. For example, the immune regulation for the control of parasitic proliferation without inducing harmful immunopathological events in the brain. Moreover, the present study provides some evidence of the immune regulation against *T. gondii* infection and protective immunity to prevent damage to host cells by an excessive inflammatory response.

The IFN-γ-mediated type 1 immune response with upregulation of the anti-parasitic factor NO is required to prevent the reactivation of *T*. *gondii* and TE in the brain (1). In the brain, microglia and infiltrating CD4 and CD8 T cells are the primary sources of IFN-γ, a cytokine leading noxious cellular effects in the CNS (1, 4). On the contrary, our earlier research also highlighted the importance of TGF-β in neuroprotection after *T. gondii* infection (8). In addition, the results of the present study suggest the importance of the interactions of various immunological factors between protective immunity and immunopathological effects by tracking of the immune response during the CI stage. First of all, changes in the immune response after *T. gondii* infection can be confirmed by microarray analysis of mRNA expression patterns, which can also predict the immunological phenotype. In the present study, there were increases in the mRNA levels of the T-cell exhaustion markers TIM3, LAG3, and KLRG1 without distinct increases in the mRNA levels of the T-cell differentiation markers TBX21, GATA3, FOXP3, and RORC during the AI stage, even though microglia activation was maintained in the M1-type. T-cell exhaustion markers are highly expressed by chronic antigen stimulation, but yet diminish the effector function of CD8 T cells (24). Accordingly, these results suggest that the inhibition of immune extension of adaptive T-cell immunity can accelerate neuronal cell death by expanding inflammatory cellular responses, although it is helpful for protective immunity of the host against *T. gondii* infection. Actually, pStat1, which is activated by IFN-γ and induces iNOS (25), was remarkably decreased at 6 weeks PI although the increase in IFN-γ expression was maintained for 12 weeks PI. Although IFN-γ-mediated activation of macrophages is critical for resistance against AI with *T. gondii, T. gondii* infection inhibits STAT1 transcriptional activity to allow the parasite to establish a CI (5, 23). Additionally, SOCS proteins, which can downregulate phosphorylation of JAK and STAT1, are actually induced by *T. gondii* infection (26). To understand the importance of microglia activation and further T-cell immune responses against *T. gondii* infection, transcript data of markers related to microglial cell polarization and T-cell-mediated adaptive immune responses were investigated. In particular, PD-L1 (B7-H1) was highly expressed in inflammatory macrophages, while PD-L2 (B7-DC) can be induced by alternative activation *via* IL-4 (27). Furthermore, PD-L1 might play a role in the downregulation of activated T cells and the expression of PD-L1 in a non-Stat1-dependent manner (27). Likewise, our results showed that PD-L1 expression was increased, as compared to PD-L2, at both transcript levels *in vivo* and *in vitro* ME-TLA stimulation (19.0–23.0- and 1.1–1.2-fold changes, respectively). At this time, M1-type activation of microglia and increases in the expression levels of the inflammatory cytokines IFN-γ, IL-12, and TNF-α were observed. However, these changes did not result in an increase in Stat-1 phosphorylation. This result provides a subtle balance for the control of parasitic proliferation and simultaneous inhibition of the increase in inflammatory cellular immune responses, which induce tissue damage during CI by altering the mRNA expression levels of PD-L1 and PD-1 because PD-1/PD-L1 interactions are required for the maturation of microglial cells and expansion of protective T-cell responses in *T. gondii* infection. However, excessive PD-1-mediated CD8 T-cell dysfunction plays a central role in immune tolerance, as well as *T. gondii* differentiation and reactivation (28–30). This is an important mechanism for the control of parasite expansion, even with the inhibition of iNOS, as in the present study.

The induction of iNOS, a M1-marker that leads to NO production, is generally elevated in the AI stage of *T. gondii* infection and in M1-like microglia (26, 31). Surprisingly, the results of the present showed that ME-TLA, a lysate antigen of *T. gondii* strain ME49, strongly induces the polarization of M1-type microglial cells and NO production. In other words, ME-TLA treatment of BV-2 microglial cells induced greater M1 polarization and NO production than treatment with IFN-γ. Simultaneously, urea production was decreased by treatment with the *T. gondii* antigens (RH and ME49) *in vitro*. TLA includes a variety of antigen pools, including TLR-based immune-triggering PAMPs (32). When soluble parasite TLA, as in the present study, stimulates dendritic cells and macrophages, the immune response resulted in TLR-signaling dependent production of IL-12 and NO (32). Accordingly, it is evident that the treatment of TLA as a PAMPs and parasite infection induce different immune triggering both *in vivo* and *in vitro*. To confirm this finding, NO production was compared between an *in vitro* infection model using *T. gondii* tachyzoites (strains RH and ME49) and an *in vitro* model using BV-2 microglial cell culture with TLA. As expected, the results were consistent with the *in vivo* infection study. NO production was decreased and mRNA expression levels of iNOS and COX-2 were lowered, while mRNA expression of Arg1 was increased by 2.9-fold. Based on these results, the authors insist that the host–parasite relationship in the regulation parasite control and host tissue damage by an excessive inflammatory response induces the control of effector molecules, which differs from the immune-triggering effect of TLA shown by the *in vitro* stimulation study. As a possible mechanism, increases in transcripts of SOCS and Arg1 during the CI stage are focused because of their immune regulatory functions.

Suppressor of cytokines signaling 1 protein is a known feedback inhibitor of IFN-γ receptor signaling and is induced directly by viable *T. gondii* parasites (26). In general, the SOCS proteins, especially SOCS1, are expressed by immune cells and cells of the CNS, and have the potential to impact immune processes within the CNS, including inflammatory cytokine and chemokine production, as well as activation of microglia (33). In this study, transcripts of SOCS1 were steadily expressed during the CI stage. It is known that SOCS induces a high Arg1:iNOS activity ratio and suppresses T-cell proliferation (34). Moreover, SOCS1 contributes to the inhibition of IFN-γ signaling and NO production without the dependency of TLR stimulation (35). In other words, due to upregulation of SOCS1 by IL-4-dependent M2 macrophage activation (34), the role of SOCS1 in the present study is sufficiently predictable for the direct increase in the Arg1:iNOS activity ratio, the decrease in transcripts of T-cell differentiation markers and Stat1 phosphorylation, and the increase in transcripts of T-cell exhaustion markers, suggesting modulations of the host–parasite relationship between parasite control and tissue damage in the brain.

Taken together, these results highlight the critical importance of M1-type microglia for the control of *T. gondii* as well as the limitation of STAT1 phosphorylation and the induction of SOCS1 to reduce detrimental inflammatory immune responses. Moreover, these results provide new insights into the characteristics of infection immunity through the host–parasite relationship during CI of *T. gondii*. In other words, *T. gondii* infection basically induces M1-type polarization of microglial cells, the limitation of IFN-γ-mediated inflammatory responses, including T-cell differentiation and Stat1 phosphorylation, and the reduction of iNOS/Arg1 ratio mediated by Socs1 induction. These phenomena are only expected by infection immunity due to the difference in the levels of effector molecules *via* the *in vitro* cellular response with TLA. In particular, this study revealed the characteristics of immune regulation in infection immunity by observing the persistent changes in the immune environment during a CI rather than at any one time.

## Ethics Statement

This study protocol was approved by the Ethics Committee of Seoul National University and conducted in strict accordance with the Guidelines for Animal Experiments (SNUIBC-R110302-1). All surgeries were performed under anesthesia and all efforts were made to ensure minimal animal suffering.

## Author Contributions

YH and E-HS conceived and designed the experiments. J-HS and YH performed the experiments. J-HS, J-PY, and E-HS analyzed the data. B-KJ, SL, and E-HS contributed reagents/materials/analysis tools. E-HS wrote the paper.

## Conflict of Interest Statement

The authors declare that the research was conducted in the absence of any commercial or financial relationships that could be construed as a potential conflict of interest.

## Footnotes

^1^http://pga.mgh.harvard.edu/primerbank/.

^2^http://pga.mgh.harvard.edu/primerbank/.

## Abbreviations

*T. gondii, Toxoplasma gondii*; PI, postinfection; CI, chronic infection; (), acute infection; NO, nitric oxide; TGF-β, transforming growth factor-β; GM-CSF, granulocyte-macrophage colony-stimulating factor; IL-12, interleukin-12; STAT1, signal transducer and activator of transcription1; SOCS1, suppressor of cytokines signaling 1 protein; MHCII, major histocompatibility complex II; TLA, *T. gondii* lysate antigen; CNS, central nervous system; TE, toxoplasmic encephalitis; iNOS, nitric oxide synthase; PAMPs, pathogen-associated molecular pattern molecules; DG, hippocampal dentate gyrus; MFI, mean fluorescence intensity; IHC, immunohistochemistry; ME-TLA, lysate antigen of *T. gondii* ME49 strain tachyzoites; (RH-TLA), (COX-2) (Arg1), lysate antigen of *T. gondii* RH strain tachyzoites.
